# Development spots in communication during the management of the intrapartum period: An interpretive multiple case study in a developing context

**DOI:** 10.4102/phcfm.v9i1.1239

**Published:** 2017-07-31

**Authors:** Doreen K.M. M’Rithaa, Susan R. Fawcus, Margaretha de la Harpe, Mikko Korpela

**Affiliations:** 1 Department of Nursing and Midwifery, Stellenbosch University, South Africa; 2 Mowbray Maternity Hospital, University of Cape Town, South Africa; 3 Department of Information Technology, Cape Peninsula University of Technology, South Africa; 4 Private, Finland

## Abstract

**Background:**

Health care activities are influenced by information communication between women during pregnancy, birth and motherhood and skilled birth attendants (SBAs) and further, between the health care workers during the continuum of care. Therefore, effective information communication processes (ICP) within and between health care facilities are a requirement for appropriate management of patients or clients. The management of the intrapartum period requires swift responses while managing critical information required for further referral and management processes. The involvement of multiple actors at different times with the same client carries the risk of communication breakdown at different points and at different levels of care. The information communicated during the intrapartum period is critical and should be accurate, timely and more importantly appropriate to enable better maternal and neonatal outcomes.

**Purpose:**

The purpose of this article is to discuss the complexities around ICP identified within a developing context that influence the management of the intrapartum period.

**Methods:**

Multi-method, multiple case study approach was used to analyse two case studies. Only the challenges from one case study (A) are discussed in this article. In-depth interviews were conducted with the SBAs. The role of observer-as-participant was utilised during the observation; field notes and document review methods were used to gather the data. Thematic analysis and activity analysis were applied to analyse the data.

**Results:**

The findings identified challenges with information and communication that influenced the management of the intrapartum period.

**Conclusion:**

This study exhibited the challenges identified as development points that can influence the management of the intrapartum period. These challenges were also identified as desirable changes from the present state depending on the perspective of the actor.

## Background

Identification of risks and abnormalities during the management of labour and birth requires effective information communication processes (ICP) by the skilled birth attendants (SBAs) within and between heath care organisations. The World Health Organization defines an SBA as:

an accredited health professional – such as a midwife, doctor or nurse – who has been educated and trained to proficiency in the skills needed to manage normal (uncomplicated) pregnancies, childbirth and the immediate postnatal period, and in the identification, management and referral of complications in women and newborns.^1,2^

Effective ICP allow for swift responses especially during labour and birth, which is the shortest period of pregnancy, yet carries the highest risk for perinatal mortality and morbidity.^1,3^ Failures in communication are the leading causes of preventable patient injuries and death particularly during labour, which places mothers and babies at risk for harm.^2,4^

Skilled birth attendants share a common goal for the outcome of labour, a healthy mother and baby, as revealed in a multicentre qualitative study involving focus groups and in-depth interviews.^4^ Major concerns arise when information communicated between the SBAs is misunderstood or misinterpreted. This increases the risk for adverse events in management of women especially during labour and birth.^4,5^ Therefore, effective ICP require the understanding of current challenges and workflows, allowing the identification of development opportunities towards seamless communication.

In a study on enhancing nurse–physician communication in obstetrics, team member behaviours were shown to influence communication^4^ and revealed that many nurses perceived the behaviour of physicians as being inconsistent with their expectations from another person in their team. These perceptions were based on the view that most physicians made decisions to quicken labour and deliver the women at a convenient time while the midwives preferred allowing labour to progress normally; they also communicated on a need to know basis, which is not what the physicians expected.

Further, the study revealed that SBAs desired respectful interdisciplinary interactions for better communication. Kindness and caring were professional traits that were valued by both parties.^4^ The midwives required understanding and patience with less experienced colleagues by the physicians while the physicians overwhelmingly preferred working with the more experienced nurses on the grounds that they had built trust and confidence in the ability of the midwives to manage labour appropriately.

Creating a culture of effective collaboration in maternity care between professional groups is increasingly seen as an essential element in good quality and safe health care. This is especially true in the context of maternity care where women don’t have straightforward labour and birth experiences and may require higher level care and transfer between settings in case of complications.^6,7^ Effective communication between health care workers requires effective collaboration enhanced by a supportive environment.^7^

Possible characteristics of effective collaboration are clear respected boundaries, effective conflict resolution systems, participation opportunities for building cohesion, accepting open and honest communication, mutual trust, and acknowledgement of interdependence and shared responsibilities. The factors that could influence effective collaboration which have an impact on effective ICP are supportive organisational structure, availability of resources, history of collaboration and a positive individual attitude.^7^ A qualitative study conducted on both midwives and doctors on inter-collaboration in the delivery suite demonstrated that ‘turf wars’ between physicians and midwives influenced communication.^8^

Information communication within and between health care settings used for decision-making during patient care relies heavily on the information collected and documented. Further, client or patient management processes are influenced by team collaboration and in-team communication within and between health care settings. The ICP during the intrapartum period are affected by the complex environment that requires swift responses in the midst of different SBAs attending to the same client within or between different health care settings. For effective management, there is need for seamless, coordinated and collaborative ICP.

The authors identified challenges with information communication within and between the midwifery obstetric units (MOUs) and the referral hospitals (RHs) during the management of the intrapartum period. The lack of or inadequate information communication influenced the coordination and collaboration of teams that require information to make decisions.

This article focuses on the challenges identified as ‘developing spots’ in ICP within the MOUs and between the MOUs and the RHs. The challenges of ICP were identified utilising an interpretive case study during the management of the woman in the intrapartum period.

## Methodology

The research adopted a multi-method qualitative multiple case study approach with embedded units of analysis in order to understand the complexities involved in the ICP within a developing country context. In this article, we discuss the challenges that emerged from one case study (A).

### Setting

The study was carried out in the Western Cape province of South Africa. The research was conducted in the Cape Town Metropole District Health Services (MDHS). The service has four intermediate level RHs and five MOUs. The MOUs manage low-risk women and are expected to refer during the perinatal period. The MOU for case study A manages approximately 150–200 births per month, while referring the high-risk women to doctors. The obstetric specialists are situated in secondary and tertiary level hospitals to manage intermediate- and high-risk women, respectively.

The focus of the study was to explore and describe the activities involved in the ICP while identifying existing challenges. For this case study, the embedded units of analysis, namely ICP within MOU A and ICP between MOU A and RH A, were analysed.

### Data collection

Data were collected through 12 unstructured interviews with SBAs (doctors and midwives), eight midwives from MOU A as well as four doctors from RH A. These interviews aimed at an in-depth exploration of the sociocultural context influencing the ICP during the management of the intrapartum period. The focus was to identify the complexities involved in the ICP. The interviews lasted between 40 and 60 min and were conducted in English as the main medium of communication among other languages utilised such as Afrikaans and IsiXhosa.^9^

The researcher assumed the role of observer-as-participant, which involved observing the ICP.^10^ The observations were confirmed by conducting short interviews with the SBAs regarding the observations. These short interviews were utilised to clarify the observations and were in addition to the in-depth interviews conducted as indicated above. The SBAs were observed as they communicated with each other utilising different means of communication such as verbal face to face, telephonic communication and written communication. Short notes were taken and the short interviews were organised with the particular SBA as soon as possible to confirm the observation. The researcher’s role was overt and the rapport created allowed the participants to freely speak about their experiences.^10,11^

Further, document analysis was done for all the written communication confirming observations and further clarifying the ICP within the MOU and between the MOU and the RHs. All the participants interviewed were female as no men were working in the two units of analysis.

### Analysis

All in-depth interviews and short interviews, which followed the observations to confirm or disconfirm observations, were transcribed. Data were analysed by thematic analysis using Atlas TI software and activity analysis. Findings were reviewed and concerns verified with the co-researchers. Themes were created in consultation with the fellow researchers through discussion and amendment after the feedback. All handwritten field notes were transcribed and coded before they were analysed.

### Ethical consideration

The research was approved by an institutional review board and the ethics committee, the Western Cape provincial department of health (protocol RP 060) and the health facilities where the research was carried out. Informed consent was obtained from each interview participant. Ethical procedures were followed according to the ethics committee recommendations.

## Results

The main themes were deduced from the activity-driven information systems development (ADISD) model.^12,13^ Understanding activities in the present state allowed the researcher to envision the complexities of ICP while anticipating development spots towards a desired state.^12,13^ The themes were deduced from the objectives while the categories emerged from the data coding and categorising process. The theme ‘challenges to ICP’ had subthemes, which included challenges with information and challenges with communication. Those are discussed in this article.

### Challenges to information communication processes

**FIGURE 1:**
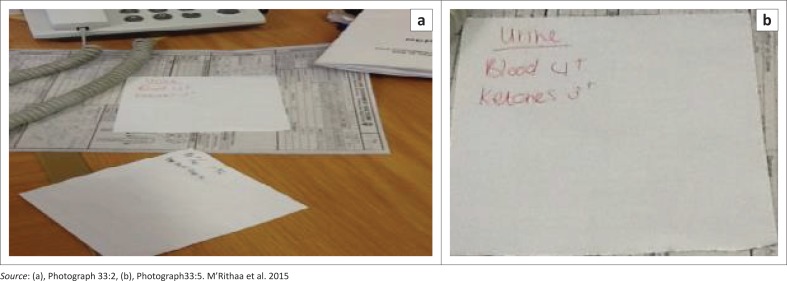
Common practice to document observations on pieces of paper.

The challenges to ICP were expressed either overtly or more covertly during the interviews and observations and had emerged as part of the culture but regarded as undesirable after further interrogation. Some of the practices observed were illuminated during the short interviews where they were not expressed as challenges but rather as desirable changes. For example, it was common practice to document observations on pieces of paper (Figure 1) with the anticipation that the information will be written at a later stage. These pieces of paper did not have the clients name on it and the SBA would try to recall whose information was documented on the said papers.

All these were pieces of papers with different patient’s observations but none of these pieces of paper had information. This led to challenges with the data collected and information used to make decisions regarding the care of women during the intrapartum period

### Challenges with information

The challenges with information emerged as inadequate information documentation, mistakes made during the documentation, challenge with the communication of information to the doctors and the fact that the computers were not used. The midwives asserted that inadequate documentation influenced the management of women during the intrapartum period. These challenges included the use of pieces of paper with the intention to transfer the information to the maternal case record at a later stage and not being able to transfer the same information on time. The result of such omissions or inaccurate data in the maternal case record resulted in inadequate communication during handovers, consultations or referrals of clients:

‘The one writes the name in there, the woman delivers then that sister doesn’t complete the rest of the form so then I just have a quick glance and see if all the columns have been completed so I would also write a note for that sister to please complete so that by the end of the month I just need it add it up.’ (Participant 2, female, midwife)

The midwives acknowledged that mistakes made during the information communication had consequences for the care of the pregnant woman such as the example below where the doctor did not make an informed decision and the woman ended up deceased, for example when a doctor perceives information communicated is appropriate and accurate and makes a decision to keep a high-risk patient at the low-risk environment based on the vital signs information communicated. Because of the distance between the MOU and RH, the doctor relies on the telephonic assessment from midwives and makes a decision based on that:

‘…. The doctor said we should discharge the woman who was pre-eclamptic. After examination of the woman, I said no man, this woman must stay here, keep her here and reassess her again. I was then busy at 13h00 my colleague came to me in a state of panic and said the woman is dead.’ (Participant 2, female, midwife)

In this case, the doctor did not make an informed decision to have the woman referred to the next level hospital. The information communicated by the midwife who was 10 km away might have been misconstrued and less attention was paid to the situation than was rather urgent. Inadequate time was seen as the reason for incomplete data or information according to the midwives. When an action was not taken, such as examining the client during the intrapartum period, the information was not transferred to the appropriate document such as a partogram. Further, the lack of or inadequate information documentation leads to inappropriate messages being communicated especially in cases where the midwives have to hand over at the end of their shift:

‘You didn’t get time to do or write the PV (per-vaginal examination) then. You say this woman is progressing very nicely but you forgot you didn’t do the observation that time so you can say exactly where the progress is supposed to be.’ (Participant 3, Female, midwife)

Listening to information communicated from the midwife allows both the sender of the information and the receiver to respect and trust the information communicated in order to act on it. The midwives expressed dissatisfaction with the information communication because of the lack of trust. This leads to friction between the midwives and the doctors in addition to reduced resources available:

‘So it’s hard to be there [near the phone] and you can hear it [the problem the midwife is presenting] and you get the doctor this side [the referral hospital] who thinks they are just sending nonsense and they get upset. They [midwives] get frustrated you know and then the doctors don’t want to take our women and then it’s the midwife who could be sending us nonsense you know it’s a case of nobody understands where the other one is coming from and I think it’s because we are overworked – both systems are stretched.’ (Participant 25, male, doctor)

Inadequate information communication seemed to increase mistrust between the doctors and the midwives. The issue of suspicion or dishonesty by the midwives was because of gaps in information when referring the patient. The patient would later be received at the RH without the information about relevant symptoms, as this vital information was only communicated verbally during the telephonic conversations.

### Communication as a challenge

When asked about the challenges the midwives faced during the ICP, two categories emerged: frustration with phones and phone calls emerged. The frustration with phone calls was viewed as inaccessible phones, which require a code or are directed through a telephone operator who does not know the state of emergency. The midwife is made to wait or ‘hang up’ the phone in order to attend to the woman labour. Further, a lot of phones in the MOUs do not work properly when the midwife needs it to consult with the doctor or refer a client to the RH. The midwives are forced to use their money and the public booth in order to call the RH for an ambulance:

‘Sometimes when we use the public phone across we use our own money you know – we use the public phone.’ (Participant 4, female, midwife)

‘Imagine? We are just stressed here because that woman won’t go there without them phoning the flying squad or whatever. Only a week or so ago we had a problem with the phones … there were no lines – the lines were dead then we had to come through to trauma to present the woman.’ (Participant 4, female, midwife)

Although the phones and calls were a problem, inappropriate information collection influenced communication during consultation processes or referrals:

‘Yes but it’s getting difficult for you now – the partogram that has not been done properly – it’s always difficult for you now because there is this space but we have to start where we have taken over and first report to the doctor that this woman was due but that they were busy and didn’t have time to do that – just because of busyness but we usually write down the word busyness and why they didn’t do it.’ (Participant 5, female midwife)

## Researcher reflections

**FIGURE 2:**
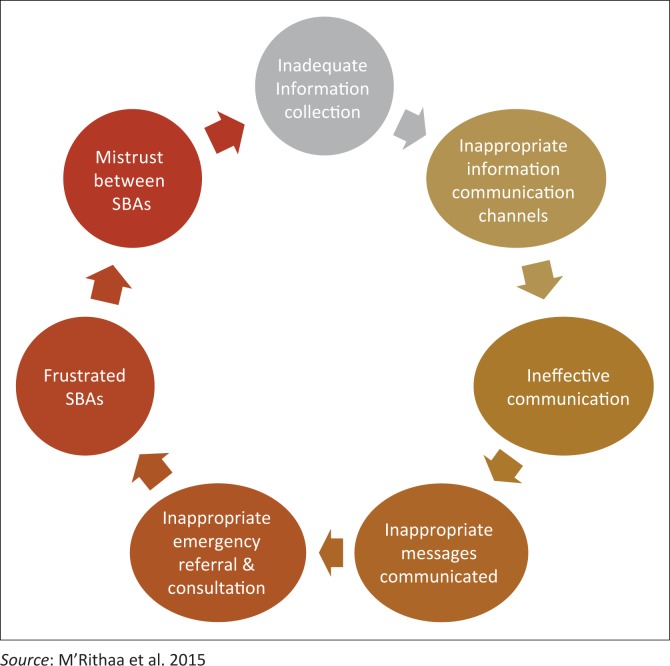
One cycle of frustration during information communication processes.

As illustrated in Figure 2, inadequate data collection influenced communication of the collected information either during handovers or consultation processes. Inappropriate information communication systems such as the flow of phone calls, unavailability of the referral doctors and unreliable tools for communication such as telephones resulted in ineffective communication.

Further, the effectiveness of the messages sent or received was influenced by the state of mind of the receiver or the sender and the language used that caused inappropriate referral and consultation processes. These inappropriate referrals and consultation processes caused frustration of the SBAs leading to mistrust and disillusioned SBAs, further causing incomplete data collection and information communication.

Further indication was that the experience and state of mind of the communicator and receiver often influenced the communication of the information or the receiving of the message communicated and documented after communication.

## Discussion

This study follows the discussion of the article published^14,15^ by the author on the actual and expected ICP during the management of the intrapartum period. The identification of challenges allowed the highlighting of development spots to improve information communication. While exploring this, the researchers were able to gain insight into the complexities associated with the communication of information during the intrapartum stage, and a myriad of challenges was identified within two realms: challenges with information and challenges with communication.

The challenges with information emerged from the fact that inadequate information had a negative influence on the care of women during the management of labour.^5^ These challenges were because of inappropriate tools for data collection, which lead to data loss, the data processes that were untimely or with unreliable sources of information.^5,6^ The acceptability of information provided, whether to the SBA in the MOU or the doctor at the RH, was based on the need to make accurate decisions according to the information presented.^6,7^ My assertion is that the challenges with data collection at the point of care had a roller coaster effect to the outcome of the intrapartum period.

The evidence from the data regarding communication challenges raised our awareness of the impact of care influenced by the communication. The actors in the communication were mostly frustrated by the processes and lost sight of the need to communicate effectively without personal influences. The lack of trust between the senders and the receivers of the messages was a main barrier to the communication.^10,11,14,16^ The midwives in the MOU felt mistrusted, whereas the RH doctors thought the midwives’ communication depended on their levels of experience.^14,15,16^ This influenced the acceptability of the message communicated whether it was during an emergency or during consultation. Further, the miscommunication affected the relationships between the midwives and the doctors leading to mistrust between the SBAs.. The SBAs felt that there was no need to provide information they believed was important as there was no trust between them. The little information communicated was further not complete as the SBAa were not assertive enough to provide the information at hand, therefore communicating inadequate information. The cycle of communication thus continued.

Notwithstanding, the challenges faced with the channels of communication, whether written or verbal, such as telephone communications, were influenced by the information collected or documented. The proposition that arises here is that identified challenges, such as inadequate information and communication, need to be elevated to development opportunities within the activities. Realigning the processes for information collection at the point of care and throughout the various activities requires considering the information tools, the data sources and the means of communication. These can be structured and protocols can be developed to bridge this gap in care.

## Conclusion

The challenges faced with the ICP were illuminated as desires for change. These challenges were related to either collection of information, documentation of the information, retrieval of the information and communication of the information. This resulted in a chain of frustration (Figure 1) among the SBAs and a sense of mistrust among them. The expression of challenges was reflected from either an optimistic or pessimistic view of reality (the half empty or half full position). Most of these challenges were thus illuminated as development spots.

The SBAs knew that they had challenges but they did not necessarily envision a desired goal state related to all the challenges. The desires that were expressed included improved phone call processes, adequate information collection and adequate communication.

Analysing the work activities in the present state allowed the visualisation of possibility for improvement of current information systems.
